# Outlines of the History of Variolous Inoculation and Vaccination, with Remarks

**Published:** 1866-03

**Authors:** S. H. Stout

**Affiliations:** Professor of Surgical and Pathological Anatomy, in the Atlanta Medical College


ATLANTA

Medical and Surgical Journal.

[New Series.]

VOL. VIIB. MARCH, 1866. [No. 1.

### ORIGINAL COMMUNICATIONS.

### ARTICLE I.

*Outlines of the History of Variolous Inoculation and Vaccination, with remarks.* By S. H. STOUT, M. D., Professor of Surgical and Pathological Anatomy, in the Atlanta Medical College.

Now that small pox is prevalent in almost every city on this continent, and there is a reasonable probability that almost every practitioner in the country will be called on to treat cases of it, and to direct the means of prevention, it is deemed desirable to present to the profession a condensed statement of what is known in regard to variolous inoculation and vaccination, compiled from the best and most recent authorities. The recent civil war has terminated, leaving many of the members of the profession in the South destitute of libraries, and too much impoverished to refurnish their shelves. If I shall succeed in presenting to them such a condensed compilation as will be useful to them, though unable to propose anything new, all will be accomplished that is now proposed.

*Origin of Variola.*

This disease was introduced into Europe from Arabia, where it is said first to have shown itself about the time of the birth of Mahomed. It is not certain that it was known to the Greeks and Romans. Prior to the discovery of the ameliorating influence of inoculation, no disease was more dreaded or more destructive of life.

*Inoculation.*

The inoculation of variolous matter, taken from a mild case of distinct small pox, as a means of ameliorating the disease, was first introduced into England by Lady Mary Wortly Montague, in 1721, from Constantinople, where she had been previously sojourning, and had witnessed its favorable result in her own child. In 1726 the royal family submitted to it, and from that time the practice was adopted throughout the civilized world, until it was superceded by the announcement of Jenner's experiments with cow pox virus.

Long prior to the above period, it is stated that the practice was in use in the South of Wales, in the Highlands of Scotland, and among the negroes of the Guinea Coast.

Our predecessors of two generations ago had better opportunities of observing and treating small pox than the practitioners of the present day; for it was often their duty to conduct whole families, or even communities, through the stages of the disease. Hospitals were set apart for the purpose. Skill in this department of practice was necessary to secure a high professional standing. The successful inoculation was necessary to avoid the patient's taking the disease in the *natural way;* for a case of small pox was in general much milder when acquired by inoculation than when produced in the natural way. The improved practice of avoiding heating remedies, and of securing free and full ventilation was universally adopted by intelligent practitioners.

After appropriate preparation, the virus was inserted under the cuticle in the same manner now in vogue, when we inoculate the vaccine virus. The lymph was prepared, and if the scab was used, it was preferred fresh. Variolous virus, as well as vaccine virus, does not retain its virtues so well in warm or moist as in dry and cool weather; for the process of putrefaction, which is destructive of its vitality, is set up more rapidly in the former than in the latter case.

This truth it is well to bear in mind when undertaking to disinfect buildings or clothing infected with small pox virus; and it accounts, in part, for the greater facility with which the disease is propagated in cool than in hot moist weather.

In choosing a subject for inoculation, if circumstances permitted an election, reference was had to the state of his health; none but the healthy were voluntarily subjected to it. Great care was paid to diet and regimen, with reference to overcoming a predisposition to inflammation. The plethoric and gross were reduced, by gentle purgatives, and even sometimes by the administration of mercurials or antimonials. Very old persons, and children under four months of age, were, if not exposed to the contagion, avoided. The cool season of the year was preferred. Very cold weather, which might necessitate the building of large fires, or the closing of windows and doors, was avoided. So, too, very warm weather was considered unfavorable to the safety of the operation.

The virus was preferably taken from young, healthy subjects, who were laboring under a very mild case of distinct small pox.

It has been my fortune to have seen and examined the scars upon the arms of many persons, who, in childhood, had undergone the operation; but few had more than one pock while suffering from the disease, and consequently only one scar, which in its characteristics resembled that following vaccinia.

*Vaccination.*

To Edward Jenner, born at the vicarage of Berkely, in Gloucestershire, in England, is due the distinguished honor of having practically first demonstrated the power of cow pox as a protective against small pox. An impression had long prevailed among the dairymen and women in Gloucestershire, that there was a certain disease, which occasionally occurred among the cows, characterized by a vescicular eruption, generally upon the udder, that if acquired by man, was a preventive of small pox. When, in 1770, he went to London as a student of medicine, Jenner frequently mentioned this popular rumor to his preceptor, John Hunter. In 1775 he gave more attention to it. Probably in 1780 he first thought of propagating the genuine cow pox by inoculating from the cow, and thence from one human being to another.

As late, however, as 1789, being unsatisfied, he inoculated his son with small pox. Having found cow pox matter in an active state on the 14th of May, 1796, James Phipps, aged eight years, was vaccinated with virus, taken from the hands of Sarah Nelmes. On the 1st of July following, the test was made by inoculating variolous matter.

In June, 1798, he published his work entitled, "An Enquiry into the causes and effects of Variolæ Vacciniæ, a disease discovered in some of the western counties of England, particularly Gloucestershire, and known by the name of cow pox."

In this work he expresses the belief that it does not originate in the cow, but is communicated to that animal from horses laboring under a disease of the heel, known as *grease*. He *suggests*, (does not prove,) that cow pox may be small pox modified by passing through the system of the cow or horse. He asserts that cow pox, once passed through the human system, leaves it secure against the infection of small pox. After experiencing much opposition, but all the while calmly pursuing his investigations, so great was the confidence of Jenner in his discovery, that in the year 1800 he expressed the opinion that "the cow pox is capable of extirpating small pox from the earth." In 1801 more than six thousand persons had been vaccinated, and tested by inoculation of and exposure to small pox. By Dr. Waterhouse vaccination was introduced into America in 1799. In 1800 it was introduced into France. It was early supported in Vienna by Dr. DeCarro, and in Milan by Dr. Sacco. In June, 1802, active vaccine lymph reached Bombay from the Persian gulf. Parliament voted Jenner £10,000 in 1802. In 1807 an additional sum of £20,000 was awarded him. Jenner died in 1823, at Berkely, in Gloucestershire. He published but little of importance subsequent to the year 1803.

(To the Cyclopedia of Practical Medicine we are indebted for the above facts; and for much of what it is proposed to insert in this paper.)

### *Of Cow Pox in the Cow.*

This disease does not frequently occur. The writer hereof recollects the case of a negro woman who contracted, in Tennessee, a disease from the cow, which was pronounced by the family physician cow pox. The negress would never take vaccinia, though the experiment was repeatedly made upon her; and though two or three times exposed to small pox, she did not contract the disease.

Cow pox is said rarely to show itself except among cattle that are herded; it then becomes epizootic. In the year 1828 the National Vaccine Board in England, after extensive enquiry, could not find it anywhere prevalent. In 1832 the disease was observed in India. In 1830 some Italian physicians found it among the cattle of the Piedmontese Alps.

It was first believed by Jenner to be a local disorder, confined to the udder of the cow. More recent observations prove it to be a general febrile disease.

"According to Jenner, the true cow pox shows itself on the nipples of the cow, in the form of irregular pustules. At their first appearance they are commonly of a palish blue color, or rather of a color approaching to livid, and surrounded by an erysipelatous inflammation. They frequently degenerate into phagedenic ulcers. The animal appears indisposed, and the secretion of milk is much lessened. The cow is subject to other pustulous sores on the nipples, which are of the nature of common inflammation, and possess no specific quality. These are free from all bluish or livid tint. No erysipelatous redness attends them. They desiccate quickly, and create no apparent disorder in the animal. Such a complaint is frequent among cows in the spring season, and when the calf is suckling. This disease is called by Dr. Jenner the spurious cow pox."

Mr. McPherson describes the disease as observed at Moorshedabad, in August 1832, as follows:

"The animals, for a day or two, appeared dull and stupid. They were seized with a distressing cough, accumulation of phlegm in the mouth and fauces, and loss of appetite. On the fifth or sixth day pustules made their appearance all over the body, especially on the abdomen, accompanied with fever and much general distress. These went on to ulceration, the hair falling off whenever a pustule ran its course. The mouth and fauces appeared to be the principal seat of the disease, being in bad cases one mass of ulceration, which impeded mastication, and proved fatal, apparently from inanimation. The mortality in this severe epizootic was calculated at from 15 to 20 per cent."

I have made the above quotations that the reader may see for himself how very different is the cow pox described by McPherson from the very mild disease described under the same name by Jenner, and from which he derived the mild and comparatively innocuous virus so valuable to the human family. The identity of the two diseases is not admissible, except on the supposition, that climate or other attendant circumstances had in the one case, added virulence to the epizootic cause. It would certainly require much experimental proof to give assurance that the virus procured from the cases described by McPherson could be safely used on the human being, or after its use would be protective against small pox.

*Casual Cow Pox in Man.*

When caught from the cow by the milkers, it is thus described: "The inflamed spots on the hands and wrists run into suppuration. The pustules assume a circular form, having edges elevated above their centre, and are of a color inclining to blue. After a time absorption takes place, and swelling appears in the axilla. Fever succeeds, accompanied with headace, vomiting, and sometimes delirium. The constitutional symptoms decline in three or four days; but the sores on the hands often remain very painful and difficult to heal. No eruption of the skin follows the decline of the feverish symptoms." (Jenner's Inquiry.)

Contrast now with the above the usual phenomena of

*Vaccinia Inoculated in Man.*

Third or fourth day: slight elevation in wound—slight redness. Fifth day: small vesicle umbilicated at top, containing transparent liquid. Sixth day: enlarged, small circle of redness at the base. Seventh day: vesicle well formed, round or oval—shining silvery appearance—areola enlarged. Eighth day: presents the appearance of a pearl set upon a rose colored circle. Areola increases in extent and redness to tenth day—vesicle becomes turgid at edges—disease now at its height; diameter of pock about one-third of an inch. Areola two or more inches in diameter—under microscope exhibiting minute vesicles upon its surface. Pock unibilicated at top; sometimes fever at or before this stage. Eleventh day: disease begins to decline. Twelfth day: scab extending over top—areola become faint—the lymph opaline, turbid and less viscid. Thirteenth day: matter purulent instead of distinct cells, the septa being broken up or absorbed, the vesicle is a single cavity. Fourteenth day: areola nearly disappeared—pock dried into a yellowish scab. After this period it gradually hardens, and during the third or fourth week separates from the skin, leaving a scar. From the eighth to the tenth day the glands in the axilla often become swollen and painful.

The above description will answer for ninety-nine in a hundred of all the cases of vaccinia in healthy subjects, and indicates that the lymph propagated by Jenner, and which is now in use in this country, is productive of a very mild disease.

*Is the virus of Vaccinia identical with that of Variola?*

Jenner contended, though he did not prove it, that small pox is a malignant form of a disease, which manifests itself in milder forms, as swine pox, chicken pox, and cow pox. This theory has found both supporters and opponents among scientific men.

"The identity of small pox and swine pox is universally admitted."—*Cyclopedia of Prac. Medicine.*

Reyer asserts that variola, vaccinia, and varicella, are modifications of one and the same disease. Does not prove it, however.

It is said that Dr. Sonderland, at Bremen, proved that cows could be infected by inhaling the effluvia of blankets saturated with variolous matter. It is stated that experiments were made in Egypt, which showed that from cows inoculated with variolous matter, a fine active vaccine virus was procured." There was no evidence furnished by these experiments, that the virus so obtained, and called vaccine, would not propagate variola.

The experiments of Mr. Ceely, of England, Dr. J. C. Martin, of Attleborough, Mass., and Dr. Basil Thiel, of Kasan, in Russia, have been often quoted as proving the identity of variola and vaccinia.

Notwithstanding careful investigators should have scrutinized the statements of these experimenters and theorists, and the proofs have never been sufficient to justify acting upon the theory of the identity of the small pox and cow pox poisons; yet during the late civil war, a medical director of a department in the Confederacy (not that of Tennessee) ordered a surgeon to inoculate a heifer with variolous matter, and use the product thereof in vaccinating soldiers. Upon an informal remonstrance by two other medical officers and myself, the order was not executed. While medical director of hospitals of the army of Tennessee, a post surgeon, of distinguished merit, notified me that having witnessed some experiments in Paris, which satisfied him that vaccinia is variola, modified by passing through the system of the cow, he had ordered the inoculation of a heifer with variolous matter, with the expectation of obtaining a benign vaccine virus for use upon the soldiers. I immediately forbade the use of the virus so obtained, and expressed my decided opinion, that the identity of the two diseases had not been proven; on the contrary, that small pox inoculated into the cow would still be contagious small pox, and as such be propagated from man to man.

*Recent Experiments.*

Recent experiments were made by Chauveau, Viennois and Meynet upon this subject; and M. Chauveau sums up in these terms the results and conclusions of these experiments in a report to the French Academy, in July 1865:

1. Human variola is inoculated on the cow and horse with the same certainty as vaccinia.

2. The effects produced by inoculation of the two diseases are entirely unlike. In the cow, variola produces merely an eruption of papules, so small that they would escape observation, if attention had not been called to their existence. Vaccinia, on the contrary, produces a vaccine eruption, the typical form of which is large and well characterized pustules. In the horse there is also a papular eruption, without secretion or crusts, produced by variola; but although this may be much more severe than that of the cow, it could never be confounded with the horse pox, so remarkable for abundance of the secretion, and the thickness of the crusts.

3. Vaccinia inoculated singly upon animals of the bovine and equine species, protects them generally from variola.

4. Variola inoculated upon the same animals generally prevents a subsequent development of vaccinia.

5. Cultivated methodically upon these same animals, that is to say, transmitted from cow to cow, or from horse to horse, variola does not approach in characters to the vaccine eruption."—[*Boston Med. and Surg. Journal.*

That vaccine is protective against small pox in a vast majority of instances, no man can deny. How and why it protects is not satisfactorily known. The theory of its identity with variola is pretty enough, and I have no remonstrance to make against any one entertaining it, if his judgment is satified; but when it is proposed to base a dangerous practice upon it, believing, as I do, that it is not thoroughly proven, I must deny it, and insist upon better proof than has heretofore been furnished.

From the quotations and statements above, the following inferences are not unfair:

1st. It is not improbable that there are several diseases which occur in the cow, which it is difficult to distinguish from each other in many cases, yet as different as small pox and varicella in the human species.

2d. Jenner himself, though he established the protective power of the lymph used by him, did not have sufficient opportunity to study the disease to the extent necessary to enable him to diagnosticate it beyond a doubt, when spontaneously occurring in the cows. Clearly he was not able to prove that cow pox always originated from the *grease* in the horse.

3d. The evidences of the identity of variola and vaccinia are so contradictory as to justify, at present, the rejection of the theory, and, consequently, the condemnation of the proposed practice to procure a new supply of lymph by inoculating the cow with variolous matter.

4th. Should the supply of Jennerian lymph become exhausted, or it should cease to prove protective, as at present informed, we know not where, with certainty, to resort for a new supply, or to find a substitute.

Practically, therefore, it is important that all civilized countries should adopt and enforce some systematic plan of preserving and propagating it in its purity.

On the continent of Europe an impression prevails to some extent, that the protective power of vaccinia is on the wane. In England this opinion is not so rife. I believe that in the United States there is now as much confidence felt in its protective power as was entertained forty years ago. May not this be due to the fact, that the American physician of intelligence has more religiously propagated the original Jennerian lymph than the European experimenter, who, confident of the certainty and accuracy of his observations, has propagated, in some instances, a cow pox not identical with that used by Jenner, and possessing less protective power?

*Can the protective power of Vaccinia be accounted for upon any other theory than that of its identity with variola?*

Why, as a rule, does small pox not occur twice in the same individual? The modern theorist may plausibly answer, that the disease produces some permanent modification in the component parts of the blood, or in their arrangement, that renders the contagious matter, or effluvia, powerless to effect those changes or movements which constitute the disease known and recognized as variola. The zymotic or catalytic agent finds not the elements necessary to sustain the fermentation, so to speak, in chemical language. Variola having wrought these supposed changes, the vaccine virus, a zymotic or catalytic agent, finds the blood destitute of certain elements necessary to the starting of the movements and changes which characterize the disease called vaccinia; or, mayhap the elements have become so combined and arranged, as to render the catalytic agent powerless to produce the necessary movements. Reverse the order of accidents above supposed, and vaccinia becomes a preventive of variola. To illustrate by a familiar example: The California yeast plant and the fungus which exists in the mass popularly termed the mother of vingar, are distinct species of the cryptogami—they both provoke the acetous fermentation; yet if in presence of one in a given specimen of an acidifiable mixture the acetous fermentation has been completed; the presence of the other will not provoke further fermentation. It is also true, that in the presence of one, the fermentation, every thing else being equal, is more active and violent than in the presence of the other. This illustration serves to convey the idea intended, which is to indicate, that if a theory to account for the protective power of vaccinia, we *must have*, one less dangerous than that in vogue can be conceived, though it may like it remain unproven.

### *A Scrap of Recent History.*

During the late civil war, in many localities throughout the South, and in the army of Tennessee, what was popularly termed *spurious vaccinia* became alarmingly frequent. My attention was first called to it as early as the middle of the year 1862; and occasional cases were reported as late as the latter part of the year 1864, though diminished in frequency. Before the appearance of these unfortunate cases, the battle of Fort Donaldson had been fought; the campaign in North Mississippi, which resulted in the battle of

Shiloh, and the final retreat of Beauregard to Tupelo, was ended; and the army, after suffering from many hardships, and the depressing effects of bad water in a miasmatic region, had moved round to Chattanooga, under Bragg, preparatory to the Kentucky campaign, erysipelas, chronic diarrhœa and chronic dysentery were prevalent. Many of the troops had not recovered from the sequelæ of measles, which had afflicted them so severely during the previous winter and spring.

Universal, and, in many instances, indiscriminate vaccination and re-vaccination was resorted to. Soldiers were vaccinated from the arms of soldiers in many instances by themselves as well as some medical officers without any care as to the normal appearance of the vesicle or dried scab used. In many cases the operation was followed by extensive erysipelas of the arm. Sometimes by phagdenic ulceration of the arm. A few lives were sacrificed, and in one instance reported to me, amputation had to be resorted to, to save the patient's life. My attention having been strangely drawn to this subject, I set about remedying it in the hospital department under my control. I forbade the use of virus obtained from the arms of soldiers or of any other persons suspected to be in bad health. Pure virus was distributed to physicians in private practice, with the request that they furnish scabs from healthy children to be used in the army. A medical officer was detailed at every hospital just to scour the neighboring country in search of healthy children on whom to propagate the virus, that a sufficient crop might be secured to avoid the necessity of using upon soldiers and citizens that obtained from adults. By this arrangement the army and the rear were supplied. The cases of *spurious vaccination* became less frequent and almost unknown in the latter months of the war.

The army and the people were at the opening of the spring of 1864, in better health than during the previous two years. No epidemics were rife, and in the hospitals during the campaign in defence of Atlanta, erysipelas and hospital gangreen were not as rife as during the previous year.

The gradual disappearance of *spurious vaccination*, so-called, is not therefore wholly attributabe to the purity of the virus. During the prevalence of a tendency to erysipelas and gangreen, it is not improbable that bad effects follow the inoculation of perfectly normal virus. Indeed a few cases were reported by reliable medical men. Many surgeons, noted for their intelligent care in the performance of every duty, report that they never had any of the bad results to follow the operation performed by them. I am well satisfied that many of the unfortunate cases, were strictly speaking, dissecting wounds, common pus in a state of putrifaction, being inserted instead of or along with the virus. And I was impressed with the idea, that the disagreeable results oftner took place in those re-vaccinated than in those who had never been subjects of vaccination. In accordance with this the advice was given not to re-vaccinate (except in cases of known exposure to small pox) those individuals who had the characteristic scar. The theoretical ground was assumed, that vaccine virus, in a patient not susceptible to the disease, might operate as common putrifying pus. A scab formed from a vesicle which has been disturbed either by accident or design probably contains much common pus, and therefore should not be used. It was therefore recommended that only those scabs shoud be used that had matured undisturbed. The usual dark mahogany colored scab, unbilicated when thorougly dry, was recognized as genuine, when taken from a healthy child who had not been exposed to an atmosphere contaminated by presence of large armies or crowded population.

The charge that spurious vaccine virus was intentionally used upon Federal soldiers is utterly without foundation. For there were more instances of unfortunate results among the Confederates.

It was not satisfactorily proven that syphilis (secondary) was transmitted by the virus. Syphilitic subjects did, in some instances, seem to have their symptoms aggravated by the artificial disease.

It was suggested by some of our most intelligent surgeons that the evil results so prevalent were due to a peculiar epidemic cause, which intensified the disease, and being gradually exhausted or expended, the phenomena of vaccinia resumed their former mild form. If the epidemic cow-pox described by McPherson, and quoted above, is admitted to be the same disease in an aggravated form as that described by Jenner, there is much plausibility in this theory.

It was contended by some surgeons that evil results never followed the innoculation of the lymph taken from the vesicle on the seventh or eigth day. Others contended that they never had evil results follow the use of the dried scab, well chosen and divested of all common pus and dead epithelial scales.

So afflicting and alarming was this *spurious vaccinia*, that an impression became rife among the soldiery that contaminated matter had been stealthily introduced among us by the Federals.

To the uncharitableness always engendered by the existence of war, is attributable the injurious impressions that were seemingly cherished on both sides of the lines, which, if founded on fact, would be disgraceful to human nature.

The pure virus. propagated as above detailed, and followed by such seemingly happy results, was obtained from the Surgeon General at Richmond, and Surgeon J. C. Mullins, who had charge of the Grant Hospital (small-pox) at Atlanta, and for a long time was Superintendent of vaccination in the department under my direction, whose observations, both in the treatment of small-pox and the propagation of pure vaccinia, were very extensive. It is to be hoped that he will at some future day place upon record the results of his experience in this regard.

### *Conclusion.*

In view of the contradictory statements above quoted, the uncertainty of the principles involved, and the intensity of interest which attaches to the subject in consequence of our late experience, and the probable prospect that the improvident, thoughtless race lately freed among us, are liable to spread the disease far and wide among the white race, and themselves to be decimated, if not like the American Indian, almost extinguished as a race. The subject of this paper becomes one of prime importance to us as scientific men and citizens. Enough has been said to prove that science has not sifted the subject to the bottom, and that the public safety requires that the public authorities should systematise vaccination, and enforce it not only for the purpose of driving out from among us loathsome and dangerous variola, but to preserve for all time pure and uncontaminated the genuine lymph, upon which as at present informed, we can alone rely with certainty to drive out from among us or ameliorate the dreadful scourge.

